# Impact of commodity terms-of-trade shocks at disaggregate level

**DOI:** 10.1371/journal.pone.0341374

**Published:** 2026-03-17

**Authors:** Rebeca Jiménez-Rodríguez, Amalia Morales-Zumaquero

**Affiliations:** 1 Department of Economics, University of Salamanca, Campus Miguel de Unamuno, E-37007, Salamanca, Spain; 2 Department of Economics, University of Málaga, Campus El Ejido, E-29071, Málaga, Spain; Southern Illinois University Carbondale, UNITED STATES OF AMERICA

## Abstract

This paper provides new evidence on the impact of country-specific commodity terms-of-trade shocks on economic growth for developing and emerging countries, not only at aggregate level but also at disaggregate level (agricultural raw materials, food and beverages, energy, and metals). Results suggest: (i) at the country group level, we find evidence supporting the so-called “terms-of-trade disconnect puzzle”; (ii) at the specific country level, the evidence is mixed (i.e., “blessing effect”, “curse effect” or “negligible effect”); (iii) at the commodity category level, it seems that output is mainly affected by shocks to the terms-of-trade for metals, followed to a lesser extent by those for energy; and (iv) statistically significant shocks occur mainly in the short run.

## 1. Introduction

The connection between commodity prices and economic activity has been extensively discussed by a large body of research. This relationship is closely linked to the behavior of commodity prices, which typically move through long-lasting cycles of boom and bust, commonly known as *commodity supercycles*. During the upswing phase, rising commodity prices tend to boost export revenues, fiscal receipts, and investment in resource-rich economies, thereby stimulating economic activity. However, prolonged dependence on these revenues can heighten vulnerability to price downturns, leading to greater volatility and persistent constraints on economic growth. In recent years, a growing body of research has focused on understanding *commodity supercycles*. Thus, for instance, [1] provide an empirical foundation for identifying long *commodity supercycles*, showing that non-oil commodities generally move in line with global demand, whereas oil prices follow a distinct pattern, thus supporting the Prebisch–Singer hypothesis of a long-term decline in the terms of trade for non-oil commodities. [2] link *commodity supercycles* to firm and region level outcomes using Brazilian data, finding that commodity booms raise employment and wages in exporting regions but also increase inequality and slow structural transformation. [3] shows that although very low-frequency commodity price movements consistent with *supercycles* exist, they account for only a small share of overall commodity price variation, with medium-term structural and policy-related factors playing a more prominent role.

From a theoretical point of view, the expected effects of an increase in the price of a commodity on economic activity differ between net exporting and importing economies of that particular commodity. Thus, when it comes to a net exporting country, the effect after an increase is expected to be positive, while a negative effect is expected for net importing countries. For a net exporting country, an increase in the price of a commodity produced there and traded in the global market is expected to increase their revenues due to the value of their exports rise relative to the one of their imports. Thus, the effect on profits and investment is positive in commodity-focused industries, but also in industries that depend on the former. For industries whose input costs increase due to higher commodity prices, the *a priori* negative effect may be more than compensated by the increase in sales if the gains from the price increase are passed on to consumers and government revenue. By contrast, for a net importing country, an increase in the price of a commodity traded in the global market implies higher production costs for industries that rely on raw materials (such as oil, metals, agricultural products, and other raw materials), but also for industries whose production depends on the input supply of the former if these pass on such an increase. Industries potentially reduce their profits and may modify their investment and production decisions. Moreover, if producers pass on higher production costs to consumers in the form of higher prices for goods and services, consumers may reduce their consumption due to either their lower purchasing power or their expectation about future prices. Consequently, investors/consumers may postpone their investment/consumption plans as they foresee a decrease in profit or income levels. Moreover, the increase in commodity prices may affect the value of imports relative to exports of the net importing country, which negatively affects its trade balance and even its competitiveness.

Empirically, there is no consensus on the existence of such a link and its sign, with the time horizon analysed being decisive. On the one hand, there is no consensus on the long-run effects of commodity prices on output, with the evidence being mixed ([4–7]; among others). On the other hand, the short-term evidence indicates a positive effect of shocks in commodity prices on economic growth in commodity-rich economies ([8]) and in less developed countries ([9–12]). It is worth mentioning that most of the empirical studies focused on the impact of commodity prices on output have considered the effects of commodities belonging to the energy category (see, for instance, [13,14]). Moreover, a related strand of research employs dynamic factor models to examine how global shocks, including fluctuations in commodity prices, are transmitted to domestic business cycles and output growth. [15] analyze the transmission of global shocks to domestic business cycles through commodity prices and world interest rates, showing that global shocks explain about one-third of business cycle fluctuations, particularly in emerging economies after 2000. [16] further find that business cycles in emerging economies tend to co-move largely due to common fluctuations in commodity prices driven by a dominant global factor. Extending this line of research, [17] show that global shocks account for more than half of GDP growth variation in small open OECD economies, although most cyclical movements stem from short-term rather than permanent shocks.

A first strand of the literature focuses on analysing the relation between commodity prices and economic growth in the long run. These studies show mixed results. In particular, several authors find no effect or a very weak effect. In this line, [4] analyses the impact of oil windfalls on six developing producer countries and concludes that much of the potential benefit to producers dissipates. [18] obtain that the impact of natural resources on Nigerian growth is limited after controlling for institutions. [19] conclude that oil windfalls contribute little to local living standards in Brazil. Finally, [8] find that long-run economic growth in commodity-rich countries does not depend on commodity prices. Other authors find positive effects of commodity prices changes on output (i.e., the so-called “blessing effect”) in the long run. For example, [20] using cross-country economic growth regressions for a set of 88 advanced and less developed countries, find that the natural resources are “blessing”. [21] obtain that natural resource abundance seems to have a positive impact on economic growth for a group of 32 advanced and less developed countries. [5] shows that a positive shock to commodity prices gives rise to a significant increase in the per capita Gross Domestic Product (GDP) for low-income countries. [22] analyse a set of commodity-rich economies and find that abundance in mineral resources has a positive influence on productivity and, consequently, on economic growth. [23] shows that the oil revenue windfall increases manufacturing activity in oil-dependent countries like Qatar. [24] finds that copper price increases originating from global demand-side shocks have a positive effect on GDP in the world’s largest copper producer (Chile), whereas price increases driven by supply-side factors or copper-specific demand shocks do not. [25] conclude that commodity prices can be considered as a leader indicator of GDP growth for G-20 countries. [26] show that commodity booms in resource-dependent countries can lead to sustained positive effects on long-term economic performance. Finally, [7] find that changes in copper and crude oil prices leads to a significant increase in the real GDP for Africa and Asia countries.

In contrast, other studies show evidence of a negative impact on economic growth after an increase in commodity prices (i.e., the so-called “curse effect”) in the long run. For instance, [27] obtain that natural resource-rich countries have never managed to achieve strong export-led or other growth. [28] show that natural resource translates into lower long-run growth in many specifications. [29] obtain a negative relationship between resource abundance and growth overall in countries with weak institutions. [30] find that countries of resource export structures are more susceptible of institutional deterioration, explaining part of the long-run negative effect on growth. [31] illustrates that resource dependence is associated with lower growth. [32] find that higher volatility of resource revenues is associated with lower long-run growth. [6] obtains that permanent increases in oil price negatively impact manufacturing output in accordance with the Dutch disease. Finally, [33] and [34] find evidence in favour of the Dutch disease effect.

A second strand of the literature analyses the consequences of commodity prices on economic growth in the short run. [9] analyse the impact of commodity-price shocks on national output and its components for Africa and obtain that increments in commodity prices induce income increases in the short run. In this line, [10] finds that higher commodity prices will be positive for African countries as exporters. [35] finds that temporary commodity prices shocks can raise short-run output through terms of trade gains. [11] obtain that commodity booms have an unconditional positive short-term impact on output for a set of less developed countries. Moreover, [12] finds short-term positive effects of commodity price booms on incomes.

Despite the vast literature on the impact of commodity price shocks on economic activity, relatively few studies have analyzed the consequences of terms-of-trade (defined as the ratio between export and import prices) shocks (which may arise from changes in export prices, import prices, or a combination of both, as pointed out by [36]) on output. Therefore, our first goal is to provide new evidence on this issue. This analysis is relevant to help policy makers and economic agents make well-informed decisions aimed at minimizing any adverse effects while leveraging any beneficial outcomes where appropriate. Notice that a terms-of-trade shock implies a sudden change in the relative prices of a country’s exports compared to its imports, which affects the purchasing power of a country’s exports in terms of its imports. Consequently, an increase in the terms-of-trade (i.e., favorable change in the terms-of-trade) can benefit a country by increasing its export revenues and improving its trade balance, while an unfavorable change can yield adverse impact on its economy. Thus, [37] and [38] obtain that terms-of-trade shocks explain more than 30% of output variance. However, [39] analyse 38 poor and emerging countries using annual data from 1980 to 2011 and find that less than 10% of movements in aggregate economic activity can be attributed to terms-of-trade shocks. [36], studying the same countries that [39] at aggregate level and considering annual data of country-specific commodity prices for the period 1980–2016, find that the nature of terms-of-trade shocks is important, and so they focus their results on the effects of shocks to export and import prices and not on the impact of the terms-of-trade shocks. While the impact of export price shocks seems to imply larger and more persistent consequences on macroeconomic variables, the effects of import price shocks are more moderate. They also obtain that up to 40% of output fluctuations is explained by export and import price shocks (taken together).

The limited existing literature examining such effects neither considers disaggregated commodity terms of trade by category nor uses country-specific commodity data. Therefore, our second objective is to illustrate how our disaggregated approach advances the previous literature. Specifically, our study goes beyond aggregate estimations by identifying how terms-of-trade shocks operate at both the country and commodity-category levels and by distinguishing between short- and long-run effects. To this end, we address three key questions. First, how do terms-of-trade shocks affect economic growth at the country level? Answering this question allows us not only to assess the effects of such shocks on each particular country, but also to identify whether there is a country or a specific number of countries that lead the results previously found in the literature ([39,36]) regarding the influence of terms-of-trade shocks on output. Second, how do terms-of-trade shocks affect economic growth at the commodity category level? Which commodity category term-of-trade shocks exhibit a greater impact on output growth? The answer to these questions allows us both to detect the specific sources of potential fluctuations in output and to determine whether there is a commodity category that acts as a leading indicator of output fluctuations. Finally, does the impact of commodity terms-of-trade shocks on economic growth occur mainly in the short run or in the long run? Knowing the answer can help policy makers to implement policies to counteract such effects if they are negative or foster them if they are positive. To address these questions, we consider, unlike previous studies, quarterly data at the aggregate and disaggregate level, country-specific commodity data and four different categories of commodities for 16 developing and emerging economies.

The rest of the paper is organized as follows. Section 2 describes the data and the econometric methodology. Section 3 presents the estimation results. Finally, section 4 includes the concluding remarks.

## 2. Data and methodology

### 2.1. Data

Country-specific aggregate commodity price indices are provided by the International Monetary Fund (IMF) Commodity Terms of Trade (CTOT) database (see https://data.imf.org/?sk=2CDDCCB8-0B59-43E9-B6A0-59210D5605D2), with technical details documented in [40]. For each economy, [40] indicate a list of up to 45 individual commodities (divided into four different categories: agricultural raw materials, food and beverages, energy, and metals) that are weighted using commodity-level trade data. Although the online database does not provide indices disaggregated by category, Bernard Gruss and Suhaib Kebhaj kindly provided them to us. For each country, this database provides three indices (export, import, and terms-of-trade indices). Each index shows two variants: (i) with time-invariant weights; and (ii) with time-varying weights.

We consider 16 developing and emerging economies (Argentina, Brazil, Bulgaria, Chile, China, Costa Rica, Hungary, India, Indonesia, Malaysia, Mexico, Poland, Russia, South Africa, Thailand, and Turkey), selected due to their increasing degree of trade openness and significant exposure to changes in international commodity prices. Table 1 lists the economies and the corresponding shares reported in the IMF dataset (World Economic Outlook, October 2020) (https://www.imf.org/en/Publications/WEO/weo-database/2020/October). For each of them, we use the global index of all internationally traded commodities as the country-specific commodity terms-of-trade index (described in the IMF CTOT database as a “*Commodity net export price index*”), in which weights individual commodity prices by their ratio of net exports to total commodity trade, using rolling, time-varying weights. In addition to the aggregate index, we also consider its main four categories (agricultural raw materials, food and beverages, energy, and metals), whose detailed composition follows [40]. The country-specific category terms-of-trade indices are calculated analogously, using the individual commodities that integrate each category weighted by the ratio of net exports to total commodity trade and with time-varying weights.

**Table 1:** Countries.

	**Shares**		**Shares**
	**(PPP)**		**(PPP)**
Argentina	0.897	Indonesia	2.065
Brazil	3.284	Malaysia	0.564
Bulgaria	0.201	Mexico	2.423
Chile	0.328	Poland	0.938
China	8.778	Russia	2.391
Costa Rica	0.063	South Africa	0.752
Hungary	0.332	Thailand	0.898
India	4.504	Turkey	1.471

*Note*: This Table shows the economies used and their shares in total world GDP at PPP, with the share for each country being the average weight of the country’s GDP in the global GDP based on PPP over the longest available sample at IMF dataset (World Economic Outlook, October 2020).

Given that our aim is to analyze the effects of country-specific commodity terms-of-trade shocks on the economic growth for each country considered at aggregate and disaggregate level, in addition to the country-specific commodity terms-of-trade index, we consider other country-specific variables such as a domestic output measure, the effective exchange rate and the consumer price.

We consider real GDP as the domestic output measure for all economies but Malaysia, where Industrial Production Index (IPI) is considered due to the lack of public data for real GDP. Most real GDP (Seasonally Adjusted) data come from FRED – Federal Reserve Bank of St. Louis. The exceptions are: (i) Russia and Thailand, whose data come from IMF-International Financial Statistics; (ii) Argentina and Indonesia, whose real GDP growth data are downloaded from OECD. In addition, for China and Thailand, we construct a proxy for real GDP by deflating quarterly current price GDP in national currency (seasonally adjusted by Tramo-Seats) with the quarterly CPI, which we use as a GDP deflator.

Aggregate Headline Consumer Price Indices (HCPI) for the developing and emerging economies are reported by the database of Global Economic Indicators (DGEI, https://www.dallasfed.org/institute/dgei). However, this database does not include the HCPI for each individual country, but Enrique Martínez-García and his colleagues at the Federal Reserve Bank of Dallas have kindly provided us the country-specific HCPI.

The real effective exchange rate (REER) (CPI-based) comes from the Bank for International Settlements for all countries considered but Costa Rica, whose data are from Bruegel data sets (https://www.bruegel.org/publications/datasets/real-effective-exchange-rates-for-178-countries-a-new-database/, version 10 February 2022).

Table 2 reports the available sample period for each economy for the variables included in levels. It is worth mentioning that we consider the fourth quarter of 2019 as the last available data in order to avoid the interference of the COVID-19 pandemic in the analysis performed. We acknowledge that this choice excludes some of the most dramatic recent CTOT shocks, such as those associated with the COVID-19 pandemic, the Russia–Ukraine war, and the United States–China trade war. This important limitation therefore represents a promising avenue for future research, in which the role of potential structural breaks could be explicitly examined.

**Table 2:** Sample period.

Country	Country-specific CTOT indicators	CPI	REER	Output	Common Sample
Argentina	1980Q1-2020Q3	1980Q1-2020Q2	1994Q1-2021Q4	1993Q2-2021Q3	1994Q1-2019Q4
Brazil	1980Q1-2020Q3	1980Q1-2020Q2	1994Q1-2021Q4	1996Q1-2021Q3	1996Q1-2019Q4
Bulgaria	1980Q1-2020Q3	1990Q5-2020Q2	1994Q1-2021Q4	1995Q1-2021Q3	1995Q1-2019Q4
Chile	1980Q1-2020Q3	1980Q1-2020Q2	1994Q1-2021Q4	1996Q1-2021Q3	1996Q1-2019Q4
China	1980Q1-2020Q3	1980Q1-2020Q2	1994Q1-2021Q4	1992Q1-2021Q3	1994Q1-2019Q4
Costa Rica	1980Q1-2020Q3	1980Q1-2020Q2	1994Q1-2021Q4	1991Q1-2021Q3	1994Q1-2019Q4
Hungary	1980Q1-2020Q3	1980Q1-2020Q2	1994Q1-2021Q4	1995Q1-2021Q3	1995Q1-2019Q4
India	1980Q1-2020Q3	1980Q1-2020Q2	1994Q1-2021Q4	1996Q2-2021Q3	1996Q1-2019Q4
Indonesia	1980Q1-2020Q3	1980Q1-2020Q3	1994Q1-2021Q4	1990Q2-2021Q3	1994Q1-2019Q4
Malaysia	1980Q1-2020Q3	1980Q1-2020Q2	1994Q1-2021Q4	1980Q1-2019Q1	1994Q1-2019Q1
Mexico	1980Q1-2020Q3	1980Q1-2020Q2	1994Q1-2021Q4	1993Q1-2021Q3	1994Q1-2019Q4
Poland	1980Q1-2020Q3	1982Q1-2020Q2	1994Q1-2021Q4	1995Q1-2021Q3	1995Q1-2019Q4
Russia	1992Q1-2020Q3	1991Q1-2020Q2	1994Q1-2021Q4	1995Q1-2021Q3	1995Q1-2019Q4
South Africa	1980Q1-2020Q3	1980Q1-2020Q2	1994Q1-2021Q4	1993Q1-2021Q3	1994Q1-2019Q4
Thailand	1980Q1-2020Q3	1980Q1-2020Q2	1994Q1-2021Q4	1993Q1-2021Q3	1994Q1-2019Q4
Turkey	1980Q1-2020Q3	1980Q1-2020Q2	1994Q1-2021Q4	1998Q1-2021Q3	1998Q1-2019Q4

*Note*: This Table shows the available sample period for each variable in levels and each country, as well as the common sample for each particular country.

### 2.2. Methodology

We consider a Vector Autoregression (VAR) model for each economy, considering the global index of all commodities for that particular country and the indices of its main four categories. This approach allows us to avoid a possible endogeneity problem.

We write the reduced form of a VAR(p) as:


$${\text{y}}_{t}^{\left(i\right)}=c+\sum_{j=\text{1}}^{p}\Phi_{j}{\text{y}}_{t-j}^{\left(i\right)}+\varepsilon_{t}^{\left(i\right)}$$ (1)

where ${\text{y}}_{t}^{\left(i\right)}$ is a (n × 1) vector of endogenous variables for country *i*; *c* is the (n × 1) intercept vector of the VAR for country *i*; $\Phi_{j}$ is the *jth* (n × n) matrix of autoregressive coefficients for country *i* and for *j = 1,2,...,p*, with *p = 4*; and $\varepsilon_{t}^{\left(i\right)}$ is the (n × 1) generalization of a white noise process with variance-covariance matrix 𝛺. In the literature analyzing the effects of commodity terms-of-trade shocks, it is common to employ a single lag when using annual data (see, for instance, [39] and [36]). Since our analysis is based on quarterly data, we standardize the specification by using four lags, which correspond to one year of dynamics, thereby ensuring comparability with studies that rely on annual frequencies.

Δ denotes changes in logarithmic values. Accordingly, we multiply the first log differences by 100 so that the resulting variations can be interpreted as quarterly percentage changes. The endogenous variables considered are: output growth ($\Delta {outp}_{t}$), growth rate of the country-specific commodity terms-of-trade index ($\Delta {cp}_{t}$), growth rate of real effective exchange rate ($\Delta {reer}_{t}$), and inflation ($\Delta p_{t}$). Thus, ${\text{y}}_{t}^{\left(i\right)}$*=* ($\Delta {outp}_{t}^{\left(i\right)},\Delta {cp}_{t}^{\left(i\right)},\Delta p_{t}^{\left(i\right)},\;\Delta {reer}_{t}^{\left(i\right)}{)}^{\prime }$.

These variables allow us to capture the different transmission channels through which CTOT shocks influence economic activity, directly through real GDP and indirectly through competitiveness and price effects.

Considering $u_{t}^{\left(i\right)}$ as a white noise vector whose variance-covariance matrix is the identity matrix (without loss of generality), the structural VAR approach considers that $\varepsilon_{t}^{\left(i\right)}$ are related to structural shocks $u_{t}^{\left(i\right)}$ throughout a matrix $A_{\text{0}}$ that satisfies $A_{\text{0}}\varepsilon_{t}^{\left(i\right)}$=${\;u}_{t}^{\left(i\right)}$. The identification is obtained by means of a Cholesky decomposition. In particular, we consider the following recursive ordering: output growth, growth rate of country-specific commodity terms-of-trade index, growth rate of real effective exchange rate, and inflation. Cholesky identification involves imposing restrictions on the contemporaneous propagation of structural shocks. Thus, variables that appear earlier in the ordering can affect those ordered later both contemporaneously and with lags, whereas variables placed later can influence the earlier ones only with a delay. In our case, output growth is placed first in the ordering, reflecting the assumption that real output does not react contemporaneously to shocks in the other variables. The growth rate of the country-specific CTOT index is ordered next. This ordering is consistent with the notion that real GDP typically adjusts with a lag to external price shocks due to the presence of real rigidities and adjustment costs (investment planning, labor market adjustments, resource reallocation, …) that cannot occur within a single quarter. Moreover, since the CTOT index is constructed at the country level, it is reasonable to assume that domestic economic conditions may exert contemporaneous effects on it. Specifically, fluctuations in output can contemporaneously affect both the demand for imports and the supply of exports, thereby influencing relative commodity prices and, consequently, the terms of trade. Inflation follows in the ordering, as it reacts contemporaneously to country-specific CTOT shocks but does not affect country-specific CTOT or output within the same period. Finally, the growth rate of the REER is placed last, since exchange rates tend to adjust almost instantaneously to both real and nominal shocks, thereby responding contemporaneously to all preceding variables.

As a robustness check, we also calculate generalized impulse response functions. The findings (available upon request) support the robustness of our baseline results.

The VAR model is estimated by maximum likelihood for each country. We calculate both the impulse responses of output growth to a country-specific commodity terms-of-trade shock and their corresponding 95% confidence bands calculated through Monte Carlo with 10.000 draws. Moreover, we consider the variance decompositions for output growth to analyze the relevance of commodity prices for output growth.

## 3. Empirical results

For the sake of clarity in the presentation of the results, the developing and emerging countries are grouped into four subgroups: Developing American countries (DAm), Emerging Eastern European countries (EEE), Emerging BRICS countries (EBRICS) and Developing Asian countries (DAs). It is worth mentioning that Brazil and India appear in two different groups of countries because we do want to explicitly analyse the BRICS economics.

Table 3 illustrates the shares of commodity imports and exports by country. The first column presents the share of total commodity imports/exports in total merchandise imports/exports as a percentage. The second column reports the percentage of total imports/exports of the four categories of commodities considered over total merchandise imports/exports. The rest of the columns display the percentage of imports/exports of each category (i.e., food and beverages, agricultural raw materials, energy and metals) over the total imports/exports of commodities considered.

**Table 3:** Commodity Imports and Exports.

	Total imports/exports of commodities over total imports/exports of merchandise (percentage)*		Total imports/exports of the 4 categories of commodities considered over total imports/exports of merchandise (percentage)**		Imports/exports of food and beverages over total imports/exports of the 4 categories of commodities considered (percentage)		Imports/exports of agricultural raw materials over total imports/exports of the 4 categories of commodities considered (percentage)		Imports/exports of metals over total imports/exports of the 4 categories of commodities considered (percentage)		Imports/exports of energy over total imports/exports of the 4 categories of commodities considered (percentage)	
	**Developing American Countries (DAm)**											
	**Imports**	**Exports**	**Imports**	**Exports**	**Imports**	**Exports**	**Imports**	**Exports**	**Imports**	**Exports**	**Imports**	**Exports**
**Argentina**	17,6	66,4	5.84	29.95	27,40	84,24	3,77	1,34	2,57	0,23	66,27	14,12
**Brazil**	25,0	65,2	11.09	27.74	18,21	68,91	3,88	2,12	1,89	5,82	75,83	23,11
**Chile**	29,4	87,0	18.45	5.09	24,99	85,46	0,70	11,00	0,81	2,16	73,39	1,18
**Costa Rica**	23,8	35,5	5.42	15.76	66,97	99,62	2,75	0,32	6,61	0,03	23,49	0,01
**Mexico**	17,4	20,0	5.01	10.39	60,68	10,68	5,59	0,29	9,58	2,89	23,95	86,04
	**Emerging European Eastern Countries (EEE)**											
**Bulgaria**	35,3	41,0	20.47	6.88	12,51	82,99	0,93	2,62	5,47	13,37	81,05	0,73
**Hungary**	18,3	12,9	8.77	3.96	9,46	80,81	1,37	1,26	9,12	2,78	79,82	15,15
**Poland**	26,5	22,2	9.68	3.80	19,83	67,68	1,96	1,77	6,40	9,34	71,69	20,71
	**Emerging BRICS Countries (EBRICS)**											
**Brazil**	25,0	65,2	11.09	27.74	18,21	68,91	3,88	2,12	1,89	5,82	75,83	23,11
**Russia**	16,7	72,4	6.36	48.62	39,47	3,33	2,83	0,14	8,18	4,67	49,37	91,81
**India**	55,2	40,8	31.64	6.91	7,84	73,08	1,26	11,29	1,20	14,33	89,66	1,01
**China**	35,8	6,9	4.52	0.94	64,60	41,49	20,13	1,06	9,07	15,96	5,97	39,36
**South Africa**	29,5	55,8	16.57	11.32	17,44	19,52	1,51	5,92	1,57	17,31	79,42	57,07
	**Developing Asian Countries (DAs)**											
**India**	55,2	40,8	31.64	6.91	7,84	73,08	1,26	11,29	1,20	14,33	89,66	1,01
**Indonesia**	33,3	58,5	12.60	37.78	34,68	22,08	8,73	8,66	5,63	4,69	50,79	64,53
**Malaysia**	29,0	32,1	8.08	15.98	32,80	32,79	10,52	6,45	9,78	3,19	46,78	57,45
**Thailand**	33,1	27,7	16.16	9.17	6,13	57,58	3,59	34,35	5,26	1,85	84,96	6,00
**Turkey**	28,6	21,3	9.70	1.70	23,51	77,06	10,93	9,41	14,43	10,59	50,93	2,35

*Note*: * Based on UNCTAD database from 2009 to 2019 (no available data for 2011 and 2012). ** Based on UN Comtrade database from the early 1990s to 2021. The shaded columns refer to the shares of imports/exports of each category over the total imports/exports of the 4 categories of commodities considered for each specific country. As an illustration, in Argentina, the 29.95% referred to the exports of the 4 categories of commodities considered with respect to the total merchandise exports is distributed as follows: 84,24% are food and beverages exports (i.e., the 25,23% of total merchandise exports are food and beverages exports), 1,34% are agricultural raw materials exports (i.e., the 0,4% of total merchandise exports are agricultural raw materials exports), 0,23% are metals exports (i.e., the 0,07% of total merchandise exports are metals exports), and 14,12% are energy exports (i.e., the 4.23% of total merchandise exports are energy exports). Finally, it is worth mentioning that Brazil and India appear in two different groups of countries because we do want to explicitly analyse the BRICs economics.

As Table 3 illustrates, DAm countries are net commodity exporters, with the categories of food and beverages and energy being the most important categories for both exports and imports. On the one hand, Argentina, Brazil, Chile and Costa Rica rely heavily on food and beverages exports (representing the 84.24%, 68.91%. 85.46% and 99.62% of the total exports of commodities considered in our analysis, respectively), while Mexico relies strongly on energy exports (with a share of 86.04% over the total exports of commodities considered). On the other hand, Costa Rica and Mexico depend considerably on food and beverages imports (with their share over the total imports of commodities considered being 66.97% and 60.68%, respectively), while Argentina, Brazil and Chile concentrate their imports mainly on energy imports (with their share over the total imports of commodities considered being 66.27%, 75.83 and 73.39%, respectively).

For the EEE group of countries, we observe that Bulgaria is a net commodity exporter, while Hungary and Poland are net commodity importers. Their commodity exports are concentrated in food and beverages, while their commodity imports are mainly energy imports.

Within EBRICS economies, there are three net commodity exporters (Brazil, Russia and South Africa) and two net importers of commodities (India and China). By categories, both imports and exports are concentrated in food and beverages and energy, although a certain degree of metals’ export is also observed.

Finally, looking at the DAs group of economies, Table 3 reveals that India, Thailand and Turkey are net commodity importers, while the opposite is true for Indonesia and Malaysia. Although it seems that exports and imports are a bit more disperse than for the other country groups, they seem to be concentrated again in the categories of food and beverages and energy.

### 3.1. Effects of country-specific commodity terms-of-trade shocks

This section shows the cumulated impulse responses of output to a 1% increase in the country-specific commodity terms-of-trade for the global index of all commodities and for the indices of its main four categories, as well as the percentage of unanticipated changes of output attributed to the corresponding commodity terms-of-trade shock at different time horizons. In particular, Table 4 presents both the contemporaneous responses and the cumulated responses up to 12 quarters after the shock for each group of countries considered, while Table 5 reports the estimated forecast error variance decomposition from 1-period to 12-period time horizons. Additionally, Tables 6 and 7 display all the country-by-country results. Notice that the country-by-country results have been grouped considering weighted averages for each group of countries. It has been established that a response is statistically significant in the grouped results when it is for at least 50% of the weighted responses in each group of countries considered.

**Table 4:** Impact of commodity terms-of-trade shocks on output.

Global	All commodities				
**Categories**		**Agricultural raw materials**	**Food and beverages**	**Energy**	**Metals**
**Developing American countries (DAm)**					
contemporaneous	0.0000	0.0000	0.0000	0.0000	0.0000
after 1 quarter	***0.0770***	−0.0539	−0.0024	0.0120	***−0.0457***
after 2 quarters	***0.1172***	−0.0811	−0.0012	0.0163	***−0.0593***
after 4 quarters	0.1142	−0.1150	0.0388	0.0093	***0.0035***
after 8 quarters	0.1333	−0.1080	0.0414	−0.0192	−0.0162
after 12 quarters	0.1424	−0.1125	0.0455	−0.0167	−0.0246
**Emerging Eastern Europe countries (EEE)**					
contemporaneous	0.0000	0.0000	0.0000	0.0000	0.0000
after 1 quarter	−0.0435	−0.0559	0.0168	−0.0216	***0.0806***
after 2 quarters	−0.0385	−0.0439	0.0753	−0.0291	0.0036
after 4 quarters	−0.0189	−0.0591	−0.0055	−0.0282	0.0486
after 8 quarters	−0.0010	−0.0698	−0.0192	−0.0211	0.0195
after 12 quarters	0.0036	−0.0502	−0.0194	−0.0109	0.0046
**Emerging BRICS countries (EBRICS)**					
contemporaneous	0.0000	0.0000	0.0000	0.0000	0.0000
after 1 quarter	0.0064	−0.0020	0.0140	−0.0049	−0.0067
after 2 quarters	0.0287	−0.0276	0.0185	0.0014	0.0057
after 4 quarters	0.0469	−0.0232	0.0477	0.0147	0.0113
after 8 quarters	0.0764	−0.0238	0.0936	0.0248	0.0161
after 12 quarters	0.0847	−0.0309	0.1141	0.0294	0.0103
**Developing Asian countries (DAs)**					
contemporaneous	0.0000	0.0000	0.0000	0.0000	0.0000
after 1 quarter	−0.0141	−0.0012	−0.0016	−0.0065	−0.0285
after 2 quarters	0.0160	−0.0088	−0.0079	0.0038	−0.0195
after 4 quarters	0.0479	−0.0139	0.0097	0.0295	−0.0152
after 8 quarters	0.0798	−0.0136	0.0602	0.0552	−0.0179
after 12 quarters	0.0832	−0.0172	0.0779	0.0568	−0.0224

Note: This Table presents the effect of a commodity terms-of-trade shock on output, which is measured as the cumulated impulse responses to an increase of a 1% in commodity terms-of-trade. Bold slanted numbers mean significance (i.e., the cumulated response is statistically significant) at the 5% significant level.

**Table 5:** Variance decomposition.

Global	All commodities				
**Categories**		**Agricultural raw materials**	**Food and beverages**	**Energy**	**Metals**
**Developing American countries (DAm)**					
1-period horizon	0.0000	0.0000	0.0000	0.0000	0.0000
2-period horizon	8.3022	3.2475	4.5411	5.3821	8.9642
4-period horizon	10.6658	4.5865	7.5481	6.5656	10.8814
8-period horizon	11.7763	5.1357	10.1701	7.5937	14.9119
12-period horizon	11.7664	5.1666	10.1244	7.6034	15.0820
**Emerging Eastern Europe countries (EEE)**					
1-period horizon	0.0000	0.0000	0.0000	0.0000	0.0000
2-period horizon	1.7300	1.7638	0.2642	1.5243	15.2794
4-period horizon	3.1027	2.1222	3.2515	2.8040	20.4816
8-period horizon	3.4921	2.7801	4.1691	2.9103	21.2099
12-period horizon	3.5402	3.0057	4.3541	3.0514	21.2314
**Emerging BRICS countries (EBRICS)**					
1-period horizon	0.0000	0.0000	0.0000	0.0000	0.0000
2-period horizon	2.7208	2.4814	1.1155	1.1514	4.3469
4-period horizon	5.6991	3.3204	2.3872	4.3714	6.5469
8-period horizon	6.7977	3.8956	4.1849	6.1744	8.2242
12-period horizon	6.9131	3.9747	4.4497	6.4083	8.3041
**Developing Asian countries (DAs)**					
1-period horizon	0.0000	0.0000	0.0000	0.0000	0.0000
2-period horizon	1.3612	2.7625	0.1583	0.7827	2.2752
4-period horizon	4.6417	3.4890	1.0279	4.4750	4.1865
8-period horizon	5.5223	3.6335	3.0500	6.1774	4.8857
12-period horizon	5.6833	3.6443	3.3745	6.4470	5.0236

Note: This Table presents the estimated forecast error variance decomposition for output at different time horizons. The entries refer to the fraction of each variable’s variance attributed to commodity terms-of-trade shocks (in %).

**Table 6:** Impact of country-specific commodity terms-of-trade shocks on output.

	Argentina	Brazil	Bulgaria	Chile	China	Costa Rica	Hungary	India
**All commodities**								
contemporaneous	0.0000	0.0000	0.0000	0.0000	0.0000	0.0000	0.0000	0.0000
after 1 quarter	−0.0421	***0.0999***	−0.2410	0.0107	−0.0068	***−0.0277***	***−0.0749***	−0.0352
after 2 quarters	−0.0561	***0.1582***	−0.0343	0.0476	−0.0123	0.0084	−0.0873	0.0293
after 4 quarters	−0.0389	0.1804	−0.0462	0.0785	0.0111	0.0351	−0.0597	0.0394
after 8 quarters	−0.0694	0.2339	0.0605	0.0605	0.0493	0.0498	−0.0309	0.0474
after 12 quarters	−0.0628	0.2444	0.0868	0.0632	0.0651	0.0469	−0.0317	0.0431
**Agricultural raw materials**								
contemporaneous	0.0000	0.0000	0.0000	0.0000	0.0000	0.0000	0.0000	0.0000
after 1 quarter	0.0128	−0.0272	0.0973	−0.0260	−0.0330	***−0.0568***	−0.0497	0.0677
after 2 quarters	0.0072	−0.0803	0.0515	−0.0774	−0.0328	−0.0301	−0.0485	−0.0042
after 4 quarters	−0.1781	−0.1130	−0.0348	−0.0314	−0.0418	−0.0456	−0.0702	−0.0313
after 8 quarters	−0.1726	−0.1026	−0.1490	0.0442	−0.0450	−0.0411	−0.0764	−0.0518
after 12 quarters	−0.1496	−0.1138	−0.0645	0.0219	−0.0494	−0.0470	−0.0825	−0.0481
**Food and beverages**								
contemporaneous	0.0000	0.0000	0.0000	0.0000	0.0000	0.0000	0.0000	0.0000
after 1 quarter	−0.0160	0.0489	−0.0161	0.0092	−0.0094	0.0086	0.0352	0.0103
after 2 quarters	−0.0323	0.0829	−0.0557	−0.0118	−0.0130	0.0338	0.0276	−0.0240
after 4 quarters	−0.0313	0.0671	−0.0687	−0.0487	0.0139	0.0568	−0.0877	−0.0004
after 8 quarters	−0.0397	0.0807	−0.0354	−0.0399	0.0759	0.0572	−0.1543	0.0620
after 12 quarters	−0.0351	0.0820	−0.0193	−0.0346	0.1139	0.0541	−0.1728	0.0669
**Energy**								
contemporaneous	0.0000	0.0000	0.0000	0.0000	0.0000	0.0000	0.0000	0.0000
after 1 quarter	−0.0398	−0.0075	−0.0788	0.0024	0.0001	***−0.0216***	−0.0292	−0.0185
after 2 quarters	−0.0188	−0.0194	−0.0690	−0.0029	−0.0004	−0.0112	−0.0332	0.0178
after 4 quarters	0.0154	−0.0203	−0.1505	−0.0137	0.0258	−0.0038	−0.0094	0.0232
after 8 quarters	−0.0247	−0.0673	−0.1315	−0.0210	0.0625	−0.0002	0.0149	0.0260
after 12 quarters	−0.0057	−0.0703	−0.0741	−0.0191	0.0754	−0.0034	0.0218	0.0238
**Metals**								
contemporaneous	0.0000	0.0000	0.0000	0.0000	0.0000	0.0000	0.0000	0.0000
after 1 quarter	0.0291	***0.0399***	0.0381	0.0071	−0.0120	***−0.0779***	***−0.2114***	−0.0654
after 2 quarters	−0.0345	***0.0599***	***0.2485***	0.0352	−0.0270	***−0.1253***	***−0.2966***	−0.0182
after 4 quarters	0.0028	***0.0889***	***0.5352***	0.0605	−0.0508	−0.1368	***−0.3885***	−0.0055
after 8 quarters	−0.0419	0.1236	0.5130	0.0496	−0.0771	−0.1511	−0.4429	0.0276
after 12 quarters	−0.0458	0.1302	0.4567	0.0506	−0.0904	−0.1539	−0.4671	0.0224
	**Indonesia**	**Malaysia**	**Mexico**	**Poland**	**Russia**	**South Africa**	**Thailand**	**Turkey**
**All commodities**								
contemporaneous	0.0000	0.0000	0.0000	0.0000	0.0000	0.0000	0.0000	0.0000
after 1 quarter	0.0285	0.0813	***0.1016***	0.0098	0.0194	***−0.0412***	−0.0459	−0.0699
after 2 quarters	0.0767	0.0707	***0.1380***	−0.0221	0.0294	***−0.0635***	0.1207	−0.0258
after 4 quarters	0.1554	0.1755	0.0880	0.0013	0.0409	−0.0545	0.1745	0.0163
after 8 quarters	0.2127	0.2691	0.0839	−0.0036	0.0539	−0.0489	0.2011	0.0274
after 12 quarters	0.2222	0.2120	0.0933	−0.0017	0.0568	−0.0468	0.1577	0.0242
**Agricultural raw materials**								
contemporaneous	0.0000	0.0000	0.0000	0.0000	0.0000	0.0000	0.0000	0.0000
after 1 quarter	0.0148	0.0955	***−0.1185***	−0.0909	0.0333	***−0.0599***	0.0370	−0.1051
after 2 quarters	0.0546	0.1891	−0.1167	−0.0627	0.0413	***−0.0957***	0.0290	−0.0674
after 4 quarters	0.1339	0.2936	−0.1075	−0.0603	0.2249	−0.1539	−0.0379	−0.1050
after 8 quarters	0.1632	0.2715	−0.1137	−0.0504	0.2421	−0.1085	−0.0063	−0.0716
after 12 quarters	0.1636	0.2119	−0.1168	−0.0357	0.2018	−0.0902	−0.0112	−0.0759
**Food and beverages**								
contemporaneous	0.0000	0.0000	0.0000	0.0000	0.0000	0.0000	0.0000	0.0000
after 1 quarter	−0.0045	0.0080	***−0.0688***	0.0172	0.0644	−0.0030	0.0064	0.0045
after 2 quarters	0.0017	0.0516	***−0.1032***	0.1201	0.1406	−0.0285	0.0023	0.0298
after 4 quarters	−0.0359	0.0033	0.0379	0.0371	0.2386	0.0379	−0.1654	0.1891
after 8 quarters	−0.0603	0.0262	0.0289	0.0322	0.2301	0.1122	−0.0475	0.2100
after 12 quarters	−0.0564	0.0427	0.0364	0.0349	0.2457	0.1215	−0.0584	0.1815
**Energy**								
contemporaneous	0.0000	0.0000	0.0000	0.0000	0.0000	0.0000	0.0000	0.0000
after 1 quarter	0.0040	0.0273	***0.0599***	−0.0067	0.0123	*−**0.0237***	−0.0212	−0.0277
after 2 quarters	0.0100	−0.0747	***0.0809***	−0.0191	0.0178	***−0.0386***	0.0346	−0.0111
after 4 quarters	0.0462	0.0311	0.0507	−0.0087	0.0225	−0.0393	0.0771	0.0177
after 8 quarters	0.0869	0.1006	0.0477	−0.0103	0.0329	−0.0477	0.0816	0.0227
after 12 quarters	0.0788	0.0271	0.0518	−0.0090	0.0335	−0.0512	0.0628	0.0234
**Metals**								
contemporaneous	0.0000	0.0000	0.0000	0.0000	0.0000	0.0000	0.0000	0.0000
after 1 quarter	0.0087	0.0342	***−0.1959***	***0.1930***	***0.0514***	0.0184	−0.1094	−0.0406
after 2 quarters	0.0399	0.0279	***−0.2411***	0.0575	***0.0878***	0.0321	−0.1203	−0.0186
after 4 quarters	0.0800	0.1400	−0.1161	0.0992	0.1565	0.0363	−0.0433	−0.0083
after 8 quarters	0.0838	0.1358	−0.2016	0.0776	0.1744	0.0628	−0.0395	0.0070
after 12 quarters	0.0940	0.1671	−0.2333	0.0748	0.1733	0.0719	−0.0041	−0.0009

Note: This Table presents the effect of a country-specific commodity terms-of-trade shock on output, which is measured as the cumulated impulse responses to an increase of a 1% in country-specific commodity terms-of-trade. Bold slanted numbers mean significance (i.e., the cumulated response is statistically significant) at the 5% significant level.

**Table 7:** Variance Decomposition by Country.

	Argentina	Brazil	Bulgaria	Chile	China	Costa Rica	Hungary	India
**All commodities**								
1-period horizon	0.0000	0.0000	0.0000	0.0000	0.0000	0.0000	0.0000	0.0000
2-period horizon	0.4229	7.1576	3.7356	0.2513	0.3158	5.3624	5.1632	3.8792
4-period horizon	1.3854	9.4399	4.9557	3.6420	0.7401	13.2324	5.4399	14.5223
8-period horizon	2.1325	11.8899	7.9279	3.9584	2.2425	13.8669	5.2610	14.6746
12-period horizon	2.1766	11.9306	8.1312	3.9344	2.5016	13.8595	5.3079	14.6625
**Agricultural raw materials**								
1-period horizon	0.0000	0.0000	0.0000	0.0000	0.0000	0.0000	0.0000	0.0000
2-period horizon	0.0393	0.4922	1.3723	0.8409	3.0933	8.6531	1.8163	3.0476
4-period horizon	1.5309	2.6074	1.5176	3.8720	2.1015	9.1578	1.7018	5.9841
8-period horizon	3.9811	2.6408	2.7754	6.4148	1.7217	9.7401	1.8254	6.2460
12-period horizon	4.1805	2.6651	3.3582	6.5387	1.6646	9.8065	1.8376	6.2531
**Food and beverages**								
1-period horizon	0.0000	0.0000	0.0000	0.0000	0.0000	0.0000	0.0000	0.0000
2-period horizon	0.1271	4.5204	0.0479	0.1556	0.2702	0.4665	0.9405	0.0987
4-period horizon	0.7947	6.1819	0.3339	1.7374	0.2448	4.7046	5.4996	2.1888
8-period horizon	1.4243	6.1369	2.7326	1.8596	2.0925	5.3187	6.1517	5.8392
12-period horizon	1.4452	6.1361	3.1247	1.9257	2.6683	5.3154	6.4809	5.8176
**Energy**								
1-period horizon	0.0000	0.0000	0.0000	0.0000	0.0000	0.0000	0.0000	0.0000
2-period horizon	0.6561	0.1023	3.2708	0.0431	0.0000	10.6211	4.2902	2.7472
4-period horizon	1.5315	0.8633	3.5158	0.2390	2.3425	12.5304	5.6404	11.7233
8-period horizon	1.7265	2.9578	3.3582	1.6906	5.6446	12.6598	5.8079	11.7629
12-period horizon	1.8623	3.0319	3.7414	1.7126	6.1478	12.7134	6.0820	11.7552
**Metals**								
1-period horizon	0.0000	0.0000	0.0000	0.0000	0.0000	0.0000	0.0000	0.0000
2-period horizon	0.1426	9.0097	0.3518	0.3934	1.4319	7.5121	11.6106	5.6671
4-period horizon	1.6893	10.5580	11.2567	7.2715	3.6629	8.7516	12.2021	8.0414
8-period horizon	2.0824	17.1118	17.1149	8.2535	4.0454	8.8508	13.3464	9.3969
12-period horizon	2.0838	17.2532	17.7126	8.1960	4.1637	8.8576	13.3631	9.3452
	**Indonesia**	**Malaysia**	**Mexico**	**Poland**	**Russia**	**South Africa**	**Thailand**	**Turkey**
**All commodities**								
1-period horizon	0.0000	0.0000	0.0000	0.0000	0.0000	0.0000	0.0000	0.0000
2-period horizon	0.3245	0.5137	13.9009	0.0855	1.4893	8.3960	0.3206	2.3048
4-period horizon	2.0930	0.4172	16.1526	1.8788	1.3025	8.3754	3.8952	3.3223
8-period horizon	2.5281	1.4962	15.6590	1.9166	1.4275	7.6227	4.5904	3.3854
12-period horizon	2.7787	1.6061	15.5659	1.9319	1.4360	7.4899	4.7431	3.3892
**Agricultural raw materials**								
1-period horizon	0.0000	0.0000	0.0000	0.0000	0.0000	0.0000	0.0000	0.0000
2-period horizon	0.3219	1.2174	8.2404	1.8289	0.6778	6.3692	0.8363	5.1095
4-period horizon	3.6120	1.8503	7.8539	2.4004	2.1913	8.2967	1.1728	5.9974
8-period horizon	5.2998	3.1397	7.7845	3.1191	7.7602	8.3876	2.0597	5.8521
12-period horizon	5.4760	3.5340	7.7318	3.3438	8.4137	8.9005	2.1512	5.8518
**Food and beverages**								
1-period horizon	0.0000	0.0000	0.0000	0.0000	0.0000	0.0000	0.0000	0.0000
2-period horizon	0.0226	0.0340	6.8807	0.0711	1.8016	0.0223	0.0071	0.0034
4-period horizon	0.4325	1.3197	12.5240	3.0801	5.1073	3.3652	3.4518	1.3903
8-period horizon	1.3853	1.4012	19.8718	3.7747	5.1668	7.0557	4.4149	2.3581
12-period horizon	1.6567	1.5522	19.7158	3.8644	5.2022	7.2938	4.5496	2.5001
**Energy**								
1-period horizon	0.0000	0.0000	0.0000	0.0000	0.0000	0.0000	0.0000	0.0000
2-period horizon	0.0141	0.1479	14.8670	0.1714	0.8806	10.4771	0.4304	0.9745
4-period horizon	0.7663	1.7545	16.8254	1.6476	0.6861	11.0544	3.4126	1.9035
8-period horizon	0.9873	3.6483	16.4862	1.7887	0.8066	9.9962	3.8829	1.9100
12-period horizon	1.0381	4.1973	16.3563	1.8309	0.8130	9.9554	4.0046	1.9253
**Metals**								
1-period horizon	0.0000	0.0000	0.0000	0.0000	0.0000	0.0000	0.0000	0.0000
2-period horizon	0.1468	0.1589	13.3111	19.7728	6.2344	4.1033	2.2004	0.7658
4-period horizon	2.1867	0.7903	14.2823	25.3867	9.2767	5.0646	1.9458	0.9840
8-period horizon	3.1150	1.0674	16.6208	24.8698	9.9143	5.7931	3.1452	1.0985
12-period horizon	3.8306	1.1803	16.9349	24.7698	9.8950	6.2612	3.2982	1.1242

Note: This Table presents the estimated forecast error variance decomposition for output at different time horizons. The entries refer to the fraction of each variable’s variance attributed to country-specific commodity terms-of-trade shocks (in %).

#### 3.1.1. DAm countries results.

The first panel of Table 4 shows that the results for the group of DAm countries (Argentina, Brazil, Chile, Costa Rica and Mexico) reveal that a 1% increase in the commodity terms-of-trade for global index of all commodities has a statistically significant positive effect on output in the short term (i.e., one and two quarters after the shock) for these countries as a group. Thus, these results suggest that a higher commodity terms-of-trade leads to an increase in output ([41,42,43]) for this group of countries that are heavily dependent on commodity exports. This evidence of a positive effect on output after a terms-of-trade shock at aggregate level gives support to the so-called “blessing effect” for the group of DAm countries. Looking at country-by-country results (Table 6), it is observed that the effect in Brazil and Mexico determines the effect of these countries as a group.

Regarding the impact of terms-of-trade shocks by commodity category (Table 4), it seems curious that the only statistically significant output response for these countries as a group is that related to the category of metals (being statistically significant negative one and two quarters after the shock and significantly positive four quarters after the shock), with the response of output to shocks to the terms-of-trade for the rest of commodities not being statistically significant. By country and category (Table 6), we observe that Brazil, Costa Rica and Mexico present some statistically significant positive or negative effect on output for different categories. In particular, a 1% increase in the terms-of-trade for metals leads to an increase in Brazilian output (“blessing effect”). In Costa Rica, we find evidence, in the very short term, in favour of the “curse effect” on output after a shock to the terms-of-trade for agricultural raw materials, energy and metals. Finally, we find evidence for Mexico in favour of the “blessing effect” for energy and the “curse effect” for agricultural raw materials, food and beverages, and metals.

Finally, we observe that shocks to the terms-of-trade for the global index, metals and food and beverages play some role in explaining the volatility of output (Table 5) for these countries as a group. At the country level (Table 7), Mexico, Costa Rica and Brazil show that the share of domestic output fluctuations attributable to the terms-of-trade for the global index is greater than 10%. By category at the country level, the volatility of output is explained by more than 10% in Brazil and Mexico for the category of metals, in Costa Rica and Mexico for the category of energy, and in Mexico for the category of food and beverages. Thus, the output variability in Brazil and Mexico seem to establish the variability of these countries as a group.

#### 3.1.2. EEE countries results.

The second panel of Table 4 reports the empirical results for the group of EEE countries (Bulgaria, Hungary and Poland). Results suggest that a shock to the terms-of-trade for the global index and for all categories but metals do not have a statistically significant impact on output for these countries as a group. For the category of metals, a shock in the terms-of-trade affects positively output in the short run (specifically, one quarter after the shock). However, having a look at country-by-country results (Table 6), we observe that there is evidence in favour of the “blessing effect” for Bulgaria and Poland after a shock to the terms-of-trade for metals and evidence of the “curse effect” for Hungary after a shock to the terms-of-trade for both the global index of all commodities and metals. Therefore, the impact in these countries as a group is determined by that of Bulgaria and Poland.

Regarding the variance decomposition, we observe that only the category of metals plays a role in explaining the variability of the output at the country (Table 7) and group (Table 5) level.

#### 3.1.3. EBRICS countries results.

The third panel of Table 4 illustrates the empirical results for the group of EBRICS economies (Brazil, Russia, India, China and South Africa). Negligible impacts of commodity terms-of trade-shocks on output are observed for these countries as a group. However, it is worth mentioning that, looking at the responses for individual countries (Table 6), shocks to the terms-of-trade for the global index of all commodities and metals have a statistically significant positive impact for Brazil (after one and two quarters and up to four quarters after the shock, respectively) and shocks to the terms-of-trade for metals show a statistically significant positive effect for Russia (one and two quarters after the shock). For South Africa, we observe that shocks to the terms-of-trade for the global index of all commodities, agricultural raw materials and energy have a statistically significant negative influence on output. In addition, a very small part of output variance is explained by the commodity terms-of-trade shocks at the country (Table 7) and group (Table 5) level, with the exception of the global index of all commodities for Brazil and India, metals for Brazil and energy for India and South Africa, where the proportion of domestic output fluctuations attributable to the terms-of-trade is greater than 10%.

#### 3.1.4. DAs country results.

The fourth panel of Table 4 reports the results for the group of DAs countries (India, Indonesia, Malaysia, Thailand and Turkey). The results indicate that shocks to the terms-of-trade of any commodities do not affect output either as a group or by countries (Table 6). Regarding the variance decomposition (Tables 5 and 7), we find again that the commodity terms-of-trade shocks explain only a very small part of output volatility as a group or individually, with the exception being the global index of all commodities and energy for India, where output variability is explained by the terms-of-trade in more than 10%.

#### 3.1.5. Summary and interpretation of results.

The results point to several key findings: (1) by groups of countries, the evidence of a significant impact of terms-of-trade shocks on output is scant. Only for the DAm group of economies, we find some evidence in favour of a “blessing effect”; (2) by country, the evidence is mixed, with results showing a positive, negative and negligible impact on output depending on the specific country and commodity terms-of-trade shock considered; (3) by commodity, shocks to the terms-of-trade for metals, followed by those for energy, seem to be the shocks that mainly affect output; and (4) when the impact of a shock to the commodity terms-of-trade is statistically significant, it occurs mostly in the short term.

It is worth noting that the finding that shocks to the terms-of-trade for metals are those that most strongly affect output may reflect the pronounced fluctuations in international metal prices since the end of 2003, which have had a significant impact on the global economy ([44]). Notice that the price of metals was relatively stable from 1995 to the end of 2003, date on which the price began to climb until the global financial crisis, where it suffered a heavy fall. This price rose considerably with the subsequent recovery until the middle of 2011, date on which the price began to decline gradually until 2016, increasing again after that date. This evolution of the international price of metals could be explained not only by demand factors (i.e., the role of China with a very high).

The results provide relevant insights into the relationship between country-specific CTOT shocks and output growth in developing and emerging economies. First, the lack of significant effects at the country-group level (except for the DAm economies) and the mixed evidence at the individual-country level suggest that structural characteristics (such as export and import composition, macroeconomic frameworks, exchange rate regimes, commodity dependence) play a crucial role. Second, the stronger and more consistent responses associated with metal and energy terms-of-trade shocks indicate that these commodities exert a particularly relevant role in driving output fluctuations, likely reflecting both their central importance in production processes and their high sensitivity to global demand cycles. However, it is worth mentioning that recent economic literature ([45–51]) highlights that the global low-carbon transition is reshaping demand patterns for the so-called “energy transition metals” – ETM (such as copper, lithium, nickel, cobalt, and rare earth elements), which serve as essential inputs for renewable technologies and electrification. This structural change in demand has significant implications for commodity prices, price volatility, and cross-commodity linkages. The mentioned studies emphasize that electrification, power grid expansion, electric vehicle production, and battery storage are increasing the intensity of metal use and putting upward pressure on metal prices, particularly when supply adjustments lag demand. Moreover, the ETM markets have become increasingly interconnected with each other and with energy markets (both shaped by geopolitical shocks and decarbonization pressures) creating new sources of volatility transmission and tail-risk amplification that traditional commodity models struggle to capture. Finally, the finding that country-specific CTOT shocks have predominantly short-run effects suggests that, while such shocks can temporarily influence output, their impact tends to dissipate relatively quickly, which is consistent with the idea that many developing and emerging economies face structural and institutional constraints that limit their capacity to transform temporary commodity windfalls into sustained economic growth.

## 4. Concluding remarks

This study provides new evidence on the effects of country-specific commodity terms-of-trade shocks on economic growth for a set of developing and emerging economies at both aggregate and disaggregate level.

We obtain several empirical regularities. First, at the country group level, we find little evidence of the impact of shocks to the commodity terms-of-trade on output, which may be partially supported by the so-called “terms-of-trade disconnect puzzle”, i.e., terms-of-trade seem to be less important in the data than in theory ([39]). It is worth noting that there is some evidence in favour of the so-called “blessing effect” for the DAm countries as a group, supporting the idea that heavily dependent commodity exporting countries are more affected by commodity shocks. Second, at the specific country level, results reveal some mixed evidence (i.e., a positive, negative and negligible impact on output) depending on the category considered. Third, when the impact of a shock to the commodity terms-of-trade is statistically significant, it occurs mostly in the short term. Fourth, it seems that shocks to the terms-of-trade for the global index and metals (followed by energy) seem to affect output, although it depends on the specific country. The fact that output is mostly affected by shocks to the terms-of-trade for metals (and to a lesser extent for energy) could be explained by the observed global economic dependence on metals and energy around the world. The global low carbon transition increases ETM demand, putting upward pressure on their prices when supply fails to adjust quickly. At the same time, ETM markets have become highly interconnected with one another and with energy market, generating new channels of volatility transmission and tail-risk amplification.

The latter empirical regularity yields several relevant policy implications. First, on the supply side, governments should promote the expansion of critical ETM (such as copper, lithium, nickel, and cobalt), while simultaneously diversifying their geographical sources of production. In doing so, they should implement friend-shoring strategies and strengthen international cooperation to reduce supply-chain vulnerabilities. Second, to strengthen macroeconomic stability and manage volatility, policymakers should seek to moderate the boom-and-bust cycles in ETM prices using structural fiscal rules or countercyclical sovereign wealth funds. To do so, it is also essential to improve coordination between metal and energy markets, given that shocks in one are increasingly transmitted to the other. Third, regarding environmental policy, the green transition must be environmentally sustainable, ensuring that renewable technologies minimize negative externalities and remain consistent with broader low-emission objectives. Finally, in terms of international governance, governments should discourage producer alliances that could distort ETM prices and instead promote cooperative frameworks for resource management. In addition, supporting sustainable development in producing countries through fair redistribution and stronger institutions would help ensure that the benefits of the energy transition are more evenly shared.
